# Applying Social Cognitive Theory in Predicting Physical Activity Among Chinese Adolescents: A Cross-Sectional Study With Multigroup Structural Equation Model

**DOI:** 10.3389/fpsyg.2021.695241

**Published:** 2022-03-15

**Authors:** Jianxiu Liu, Muchuan Zeng, Dizhi Wang, Yao Zhang, Borui Shang, Xindong Ma

**Affiliations:** ^1^Division of Sports Science and Physical Education, Tsinghua University, Beijing, China; ^2^Vanke School of Public Health, Tsinghua University, Beijing, China; ^3^Department of Kinesiology, Hebei Institute of Physical Education, Shijiazhuang, China; ^4^Department of Social Sciences, Hebei Sport University, Shijiazhuang, China

**Keywords:** social cognitive theory, physical activity, Chinese, adolescents, structural equation model

## Abstract

This cross-sectional study aimed to assess the applicability of social cognitive determinants among the Chinese adolescents and examine whether the predictability of the social cognitive theory (SCT) model on physical activity (PA) differs across gender (boys and girls) and urbanization (urban and suburban). A total of 3,000 Chinese adolescents ranging between the ages of 12–15 years were randomly selected to complete a set of questionnaires. Structural equation modeling (SEM) was applied to investigate the relationships between social cognitive variables and PA in the urbanization and gender subgroups. The overall model explained 38.9% of the variance in PA. Fit indices indicated that the structural model of SCT was good: root mean square error of approximation (RMSEA) = 0.047, (root mean square residual) RMR = 0.028, goodness of fit index (GFI) = 0.974, adjusted goodness of fit index (AGFI) = 0.960, Tucker–Lewis coefficient (TLI) = 0.971, and comparative fit index (CFI) = 0.978. Regarding the subgroup analysis, social support (critical ratios [CRs] = 2.118; *p* < 0.001) had a more substantial impact on the PA of adolescents in suburban areas than that in urban areas, whereas self-regulation (CRs = −2.896, *p* < 0.001) had a more substantial impact on the PA of adolescents in urban areas than in suburban areas. The results indicate that the SCT model predicts the PA of Chinese adolescents substantially. An SCT model could apply over a range of subgroups to predict the PA behavior and should be considered comprehensively when designing interventions. These findings would benefit PA among the Chinese adolescents, especially across genders and urbanization.

## Introduction

Regular physical activity (PA) is essential for the health of adolescents, as it reduces the risk of chronic diseases, obesity, and mental health problems and improves cognitive health indicators (Kruk, 2007; Lipnowski et al., 2012; Poitras et al., 2016). Despite the considerable evidence showing the benefits of PA, 80.3% of adolescents (13–15 years old) failed to meet the recommendation of 60 min of PA daily (Hallal et al., 2012). Physical inactivity among adolescents is widespread worldwide (Sisson et al., 2010; Patnode et al., 2011). In 2016, adolescents aged 11–17 years had a prevalence of physical inactivity of 81.0% (77.6% of boys and 84.7% of girls) globally (Guthold et al., 2020). According to a 2017 survey in China, only 34.1% of children and adolescents met the recommendation of 60 min or more of moderate to vigorous PA (MVPA) per day (Zhu et al., 2019). Evidence indicates that the habits of PA behavior established in adolescence are likely to track into adulthood (Patnode et al., 2011; Hayes et al., 2019). Therefore, engaging in PA during this period is particularly important for the whole life span of the individual.

Evidence suggests that interventions using health behavior theories would effectively change the population-level behavior in “real world” contexts (Hagger and Weed, 2019) and may help in explaining the maintenance of health-related behaviors (Kwasnicka et al., 2016). Furthermore, health behavior theories provide an effective framework to understand the mediators and moderators of the behavior. Bandura (1986) proposed that human behavior, personal factors (such as, cognition), and environmental factors are affected by each other within a framework of reciprocal determinism, termed as triadic reciprocal causation. Social cognitive theory (SCT) represents a causal model in which the self-efficacy is set to the influence human behavior directly and indirectly *via* other mediating processes that include outcome expectations, social support, and self-regulation (Bandura, 1991, 1998, 2004; Bandura et al., 1997). Self-efficacy reflects the judgment of own ability of an individual to accomplish a specific health behavior. Outcome expectancy reflects the individual’s perception of the likely social, physical, self-evaluation outcome of completing a specific health behavior. Social support is the perceived support for health behaviors from important others, such as family and friends. Self-regulation operates through a set of psychological subfunctions to influence the health behavior (e.g., self-monitoring, judgmental, and self-reactive influences).

Key SCT determinants of PA include social supports, self-efficacy, outcome expectations, and self-regulation of participating in PA (Bandura et al., 1997; Anderson-Bill et al., 2011a; Ramirez et al., 2012). In previous studies, SCT indicates that social and psychological determinants influence the behavior (Bandura, 1986, 1998) and that self-efficacy is the most relevant determinant to PA in SCT among all key determinants (Dewar et al., 2013; Ah Hong et al., 2017; Beauchamp et al., 2019). Researchers have used SCT to predict the PA behavior in different countries (e.g., the United States, Australia, and Iran) (Gao, 2012; Dewar et al., 2013; Bagherniya et al., 2015). The SCT has been widely applied to explain and change the PA behavior across a wide range of age groups and proved successful. Researchers found that the SCT serves as a good framework for researchers studying health promotion and PA in the parents of African American children (Webber-Ritchey et al., 2018). Furthermore, SCT was proved to be effective when used in the intervention in persons with multiple sclerosis and breast cancer (Auster-Gussman et al., 2021; Silveira et al., 2021). The overall predictability of SCT ranged from 5 to 52% among adolescents (Taymoori et al., 2008; Lubans et al., 2011; Martin et al., 2011). However, there are not many pieces of research that use SCT to predict the PA behavior in Chinese adolescents.

The positive effects of social-cognitive determinants on PA prompted researchers to examine the invariance of SCT and the differences between the subgroups of different demographic variables. Identifying the differences in the predictive power of the SCT model and its key determinants among the demographic subgroups is essential for improving our understanding of PA behavior and designing effective interventions. One study reported the predictive power of SCT on PA among urban and underserved middle school students and found that the overall SCT predictability was 19% in urban areas and 12% in underserved areas (Martin et al., 2011). There are numerous gaps in the levels of PA between adolescents living in urban and suburban areas. These differences can be partially explained by socioeconomic status, environment/equipment barriers, social support, and other factors (Felton et al., 2002; Hartley, 2004). However, few studies have compared the difference of social-cognitive determinants in urban and suburban areas. Additionally, the predictive power of SCT’s determinants has been analyzed in several research studies. Self-efficacy, parental social support, and friend social support showed no differences between boys and girls (Martin and McCaughtry, 2008). In contrast, boys are more physically active when a parent praises them for being physically active (Ah Hong et al., 2017). Therefore, the predictive power of SCT and its determinants for PA in different gender groups should be further confirmed.

In terms of the estimates of invariance, few studies have confirmed the invariance of SCT across different gender or community subgroups (Beauchamp et al., 2019). Examining the invariance is important to ensure that any differences reported in communities or genders are not merely a function of differences in interpretation of the measures. There is a possibility that the responses to items may be influenced by gender or region of respondents (Duda and Hayashi, 1998). Investigating factorial invariance across the country, age, or gender subgroups allows the examination of the construction of questionnaires, which may result in the measurement of a latent construct similarly across samples. Furthermore, a majority of previous research studies focus on adolescents from western countries. People of Eastern cultural backgrounds have been long overlooked. Therefore, the present study aims to (a) assess the generalizability of the SCT measurements among the Chinese adolescents, (b) test the invariance of the SCT model across gender and urbanization, and (c) examine whether the predictive power of the SCT model on PA differs across the gender (boys and girls) and community subgroups (urban and suburban).

According to evidence provided by previous studies in other countries (Dewar et al., 2013; Ah Hong et al., 2017), we hypothesize that (1) SCT could predict the PA behavior of Chinese adolescents, and that (2) there are no differences in the predictive power of SCT on PA behavior in different gender subgroups. Moreover, previous studies reported numerous gaps in PA levels among adolescents living in urban and suburban areas (Felton et al., 2002; Hartley, 2004). Support from parents and social community members might differ between these two subgroups (Ma et al., 2019). Thus, we hypothesize that (3) social support and self-regulation would differ in urban and suburban subgroups to predict PA.

## Materials and Methods

### Design and Participants

A cross-sectional survey was conducted in three cities in China (Beijing in the northeast, Shanghai in the southeast, and Urumchi in the west) from March to May 2018. A total of 3,000 students ranging between 12 and 15 years were randomly selected from local middle schools in each city to complete the questionnaires. The sample size in a structural equation modeling (SEM) study is usually 10 times the number of variables according to the rule of thumb. Thus, the sample size is sufficient for this study. The exclusion criteria are as follows: (a) those who were not between 12 and 15 years old; (b) those who were unable to participate in normal physical activity in the previous week due to injuries or sickness; and (c) those who completed less than 50% of their questionnaire. In total, 2,502 valid questionnaires were obtained, and the validity rate of the questionnaire was 83.4%. Missing values in the dimensions of social-cognitive variables were imputed with mean values of each dimension.

### Procedure

Simple random sampling was used in the study. First, all the schools in these cities were coded. A random number generator was used to randomly select 18 schools, 11 of them from urban areas and 7 from suburban or semirural areas. Second, the teachers in each selected school were contacted, and the purpose of the study was explained to acquire the permission for contacting their students. With approval, the survey was conducted in the classroom setting, and participants were assured that the participation was voluntary and that they were free to withdraw at any time. Before administering the survey, we explained that it was not a test and that we were interested in the actual PA and social cognitive status of participants. All answers were confidential, and all identifying information was kept anonymous to minimize the risk of social desirability bias. Participants were reminded to complete all the questions within 15–25 min. Informed consent was obtained from each student and their parents. Ethics approval to conduct the study was obtained from the ethics committee at the host university.

### Instruments

Participants were asked to fill out their demographic information (e.g., age, gender, and living area), SCT-related psychological scales, and the PA questionnaire.

*Self-efficacy* was measured by using the Self-efficacy for Exercise Scale (SES). SES was used to test the situational confidence in persisting with exercising (Benisovich et al., 1998). The head of the scale reads, “please evaluate how much confidence you have when participating in regular PA during your leisure time.” The 28-items SES uses a five-point Likert-type scale ranging from not confident at all (1) to completely confident (5), and consists of six dimensions (Norman, 1998): (a) negative effects, (b) resistance from others, (c) making excuses, (d) bad weather, (e) exercising alone, and (f) inconveniency. A sample questionnaire item was “I’m tired.” This scale has been proven to have good validity and reliability in assessing psychological problems among Chinese adolescents (Cronbach’s alpha = 0.91) (Yin, 2007; Ren et al., 2020). The reliability test of the scales in the present study is α = 0.93.

*Self-regulation* was measured by using the Exercise Goal-Setting Scale (EGS) and the Exercise Planning and Scheduling Scale (EPS) (Weinberg, 2002). A five-point Likert scale from 1 (“does not describe”) to 5 (“describes completely”) was used for scoring both the EGS and EPS (10-items each) (Rovniak et al., 2002). The EGS and EPS have been proven to be valid and reliable in assessing psychological problems among Chinese adolescents (Cronbach’s alpha = 0.88) (Zhao, 2008; Xu et al., 2017). The head of the scale reads, “please evaluate whether the following descriptions are consistent with your when participating in PA.” A sample item was “I always schedule a week’s exercise time in advance.” The reliability test of the scales in the present study is α = 0.91.

*Outcome expectation* was measured by a scale developed by Lewis for the Physical Activity for Risk Reduction study and was further amended by Lewis et al. (1993) and Rogers et al. (2007). Participants were asked to rate their agreement on statements that include 12 psychological, social, or physical benefits on a five-point Likert scale (1 = strongly disagree to 5 = strongly agree). The scale has proven good validity and reliability in assessing psychological problems among Chinese adolescents (Cronbach’s alpha = 0.94) (Xu et al., 2020). The head of the scale reads, “please select the appropriate option and feeling when you participate in regular PA.” A sample item was “Regular exercise will increase my muscle mass.” The reliability test of the scales in the present study is α = 0.90.

*Social support* was measured by a scale constructed by Sallis et al. (1987). The items described supportive actions and words that encourage PA. Subjects were asked to rate family and friends’ frequency to perform as the items described. The scale has been proven to have good validity and reliability among the Chinese and American children and adolescents (Hsu Y. W. et al., 2011; Liang et al., 2014). The scale used 12 items for family members and 10 items for friends on a five-point scale ranging from 1 (never) to 5 (always). The head of the scale reads, “please rate what your family (e.g., parents, siblings, and grandparents) and friends (e.g., close friends, acquaintances, classmates) have said and done in the past 3 months concerning PA.” An example item is “Offer to exercise with me.” The reliability test of the scales in the present study is α = 0.853.

*Physical activity* was measured by the PA questionnaire for adolescents (PAQ-A) developed by Kowalski et al. (2004). The PAQ-A is a self-administered, 7-day recall instrument. It was developed to measure the general levels of PA for adolescents. The questionnaire classifies PA of adolescents into different activity levels and investigates the relationship between PA and health outcomes (MacKelvie et al., 2001). A five-point Likert scale from 1 (“Never”) to 5 (“Above six times a week”) was used to measure that how many times subjects participated in PA in a whole week. PA level is classified as low (≤2), moderate (>2 and ≤3), and high activity (>3), according to the mean score of nine items (Chen et al., 2008). An example question is, “In the past 7 days, how many days have you exercised after school (not including weekends)?” PAQ-A has been proven to have good validity and reliability in assessing psychological problems among adolescents (Voss et al., 2017).

### Data Analyses

Descriptive statistics (i.e., means and SD) were computed using Stata 15.0. SEM was performed by AMOS 25.0, and the model was estimated using the maximum likelihood techniques to investigate the relationships between social cognitive variables and PA in urbanization and gender subgroups (Byrne, 2010). First, a confirmatory factor analysis (CFA) was conducted considering the latent variables and observed variables following Yuan et al.’s method (Yuan et al., 1997). A CFA was conducted to test the validity of the scale and the relationship between the latent variable. All standardized factor loading within this single factor should be larger than 0.5 and statistically significant. Model fit was assessed using root mean square error of approximation (RMSEA), root mean square residual (RMR), comparative fit index (CFI), normed fit index (NFI), incremental fit index (IFI), and goodness of fit index (GFI). The accepted cut-offs for the values of CFI, NFI, IFI, and GFI should be greater than 0.90; the thresholds for RMSEA and RMR should be less than 0.05.

Second, the overall fit of the resultant models was computed to assess the predictability of SCT among the Chinese adolescents. A number of the goodness of fit indices representing absolute, comparative, and residual aspects of fit were used, specifically chi-square (χ^2^), degree of freedom (*df*), RMSEA, RMR, GFI, CFI, adjusted goodness of fit index (AGFI), Tucker–Lewis coefficient (TLI), parsimony goodness of fit index (PGFI), and parsimony normalized fit index (PNFI). The RMSEA and RMR of less than 0.05 indicate excellent model fit (Loehlin and Beaujean, 2016). The GFI, AGFI, TLI, and CFI greater than 0.9 are excellent. PGFI and PNFI greater than 0.5 are excellent (Bollen and Long, 1993).

Third, the invariance analysis across groups is a logical prerequisite for conducting the multigroup comparisons (Vandenberg and Lance, 2000). Invariance models within the pattern of relationships among theoretical constructs (i.e., covariances) and the latent mean difference were estimated to compare if the SCT operates equivalently across the gender and urbanization subgroups. The differences between the groups can be evaluated by examining differences between the models that assume equalities among the parameters with models. A theoretical model is separately applied to each subgroup, and then the invariance analyses are conducted. Before the invariance models can be estimated, it must be established that a model without any invariances (i.e., a model that is different in each group) is reasonable. This model can be used as a basis of assessments for more constrained models. Thus, Model 1 represents unrestricted model (non-invariant, unconstrained model); Model 2 represent measurement equivalent model: (identical factor loading across the subsamples); Model 3 includes Model 2 constraints plus equal factor variances and covariances; Model 4 including Model 3 constraints plus equal paths; Model 5 includes Model 4 constraints plus equal factor residuals (“fully constrained”). Models 4 and 5 refer to the latent construct level, which deals with more substantive information about how subsamples may differ and are similar. The chi-square difference test and the TLI were applied to test the equality constraints to compare the models. If the χ^2^ difference is statistically significant, then evidence of cross-group inequality exists. However, the χ^2^ difference test would be too strict for a large sample study (Quintana and Maxwell, 1999). TLI estimates the models for the groups separately and sums the chi-squares and the degrees of freedom. A difference of more than 0.05 in TLI or 0.01 in CFI is considered trivial in practical terms (Cheung and Rensvold, 2002; Nigg et al., 2009). To complete the final research objective (i.e., determining the explained variance and comparing the strength of regression paths of the SCT constructs in predicting PA across democratic groups), pairwise critical ratios (CRs) for differences between parameters were examined to determine if there were significantly different regression paths for the demographic groups. A CR value larger than 1.96 indicates statistically significant differences in the latent mean.

## Results

Table 1 demonstrates the characteristics of the participants. A total of 2,502 adolescents had a mean age of 13.26 (*SD* = 0.87). Most of the participants were 13 or 14 years old; 779 (31.1%) individuals were from Beijing, 388 (15.5%) were from Shanghai, and 1,335 (53.4%) were from Urumqi. Of all the participants, 48% were boys, and 52% were girls. Meanwhile, 58.6% of the participants were from urban areas, and 41.4% were from the suburbs.

**TABLE 1:** Descriptive statistics of participants.

Category	Demographic characteristic	Sample size	Percentage
City	Beijing	779	31.1%
	Shanghai	338	15.5%
	Urumqi	1335	53.4%
Urbanization	Urban	1467	58.6%
	Suburban	1035	41.4%
Gender	Male	1191	48.0%
	Female	1290	52.0%
Age	≤12	276	11.2%
	13	1381	56.1%
	14	656	26.7%
	≥15	147	6.0%

### Evaluation of the Measurement and Structural Models

#### Measurement Model

A test of the measurement model indicated a highly satisfactory fit to the data in all five measures: RMSEA = 0.047, CFI = 0.980, NFI = 0.977, IFI = 0.980, and GFI = 0.976. All the factor loading of self-efficacy, self-regulation, outcome expectation, and social support ranged from 0.50 to 0.95 (*p* < 0.001), which confirmed the convergent validity of the indicators (Anderson and Gerbing, 1988). Thus, the measurement model was used to test the hypothetical structural model (the figure attached in Appendix A shows the validity of dimensions).

#### Structural Equation Model

**FIGURE 1:**
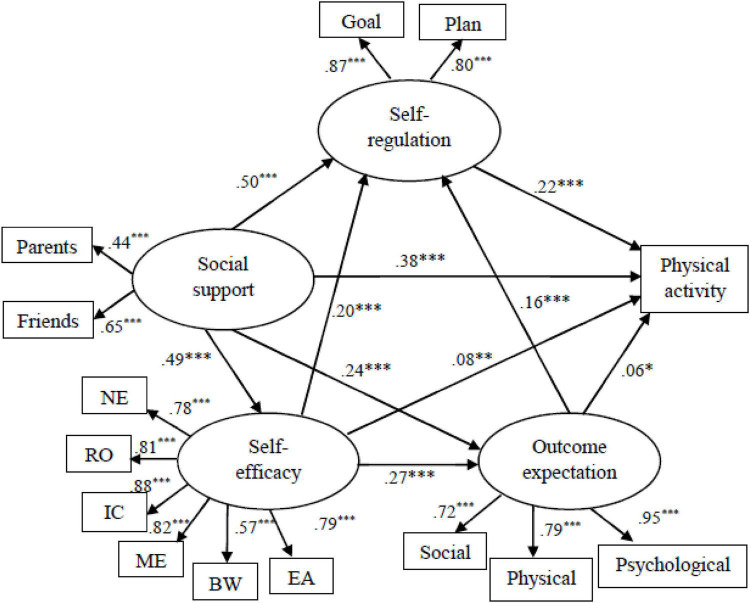
The structural equation model of the overall sample. NE, negative effects; RO, resistance of others; ME, making excuses; BW, bad weather; EA, exercising alone; IC, inconveniency. * indicates *p* < 0.05, ** indicates *p* < 0.01, *** indicates *p* < 0.001.

The structural model was tested with all the paths depicted in Figure 1. Fit indices indicated that the structural model was good: RMSEA = 0.047, RMR = 0.028, GFI = 0.974, AGFI = 0.960, TLI = 0.971, and CFI = 0.978. The overall model explained 38.9% of the variance in PA.

Direct effects, indirect effects, and the portion of total effects mediated by other variables reflect how SCT variables influence PA (Table 2). The total effect of a latent variable on PA is the combination of direct and indirect effects. Social support had the greatest total effect on PA (β_*total*_ = 0.583, *p* < 0.001). Higher levels of social support led to the higher levels of self-efficacy, outcome expectations, and self-regulation. Self-regulation exerted a moderate total effect on PA (β_*total*_ = 0.221, *p* < 0.001). Higher levels of self-regulatory skills, such as goal setting and planning, directly resulted in the higher PA levels. Self-efficacy had a small total effect on PA (β_*total*_ = 0.153, *p* < 0.001). Outcome expectations exhibited a small total effect on PA (β_*total*_ = 0.093, *p* < 0.05).

**TABLE 2:** The direct, indirect, and total effects of the variables in the social cognitive theory (SCT) model.

Latent variables	Direct effects	Indirect effects				Total effects			
			
		SE	OE	SR	PA	SE	OE	SR	PA
Social support	0.38***	–	0.131***	0.159***	0.207***	0.486***	0.375***	0.656***	0.583***
Self-efficacy	0.08**	–	–	0.044***	0.070***	–	0.269***	0.246***	0.153***
Outcome expectations	0.06*	–	–	–	0.036***	–	–	0.162***	0.093***
Self-regulation	0.22***	–	–	–	–	–	–	–	0.221***

NE, negative effects; RO, resistance of others; ME, making excuses; BW, bad weather; EA, exercising alone; IC, inconveniency. * indicates p < 0.05, ** indicates p < 0.01, and *** indicates p < 0.001.

#### Multigroup Analysis

Multigroup SEMs were performed to examine the invariances of the SCT model across the multigroup and whether SCT operates equivalently in regression paths in predicting the PA across gender (boys and girls) and urbanization (urban and suburban).

##### Urbanization

**FIGURE 2:**
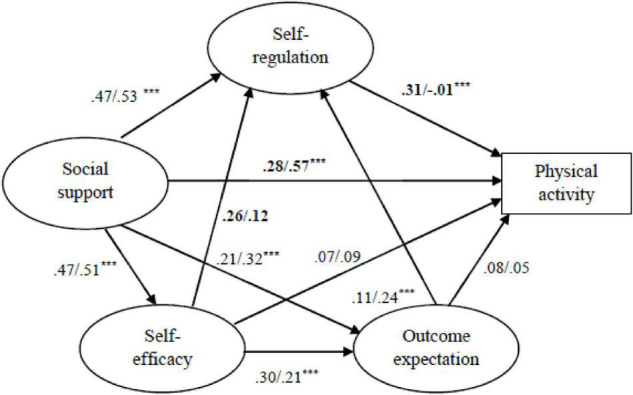
The multigroup structural equation modeling (SEM) across urban and suburban. *** indicates *p* < 0.001, otherwise *p* > 0.05. Bold indicates significant differences of the path coefficients (critical ratios [CRs] > 1.96).

The SEM was tested in urban and suburban adolescents. In both groups, the hypothesized model is a good representation of the data, as shown in Table 3. Given that the model results in good fit indices for urban and suburban groups, a multigroup SEM with latent variables was conducted to explore which parameters could be invariant across the urbanization. Table 4 presents the constrained models (Models 2–5) and the different tests of these models with the unconstrained model (Model 1). The RMR, GFI, AGFI, TLI, CFI, and RMSEA indicate excellent model fit. By examining the differences between the constrained and unconstrained models, all models appear to be significantly different in any case at the 1% level. The TLI and IGF indices suggest negligible differences between Models 2, 3, and 4 to the unrestricted model. Thus, Model 1, the unrestricted model, will be used to assess the path coefficients in the two groups (Figure 2). The architecture of the social-cognitive variables on PA was tested to see differences between the urban and suburban groups. After comparing CRs for differences between parameters, social support (CR = 2.118; *p* < 0.001) had a more substantial impact on the PA of adolescents in suburban areas than that in urban areas, whereas self-regulation (CR = − 2.896, *p* < 0.001) had a more substantial impact on the PA of adolescents in urban areas than in suburban areas.

**TABLE 3:** The goodness of fit index (GFI) of urban and suburban groups.

	*n*	RMSEA	RMR	GFI	AGFI	TLI	CFI	PGFI	PNFI
Urban	1467	0.046	0.027	0.973	0.959	0.973	0.980	0.630	0.728
Suburban	1035	0.044	0.029	0.974	0.957	0.973	0.981	0.593	0.684
									

**TABLE 4:** Nested Models of Multigroup SEM across urban and suburban.

Model	Chi^2^	df	Chi^2^/df	*p*	RMR	GFI	AGFI	TLI	CFI	RMSEA	Δ Chi^2^	Δ *p*	Δ TLI
**Urbanization**													
Model 1	522.45	136	3.20	<0.01	0.03	0.96	0.94	0.97	0.98	0.03	−	−	–
Model 2	549.89	149	3.06	<0.01	0.04	0.96	0.94	0.97	0.98	0.03	27.44	0.01	<0.01
Model 3	567.14	155	3.09	<0.01	0.04	0.96	0.94	0.97	0.98	0.03	44.69	< 0.01	<0.01
Model 4	574.21	159	3.07	<0.01	0.04	0.96	0.94	0.97	0.98	0.03	51.76	< 0.01	<0.01
Model 5	647.98	173	3.27	<0.01	0.04	0.95	0.94	0.97	0.97	0.03	125.53	< 0.01	<0.01

##### Gender

**FIGURE 3:**
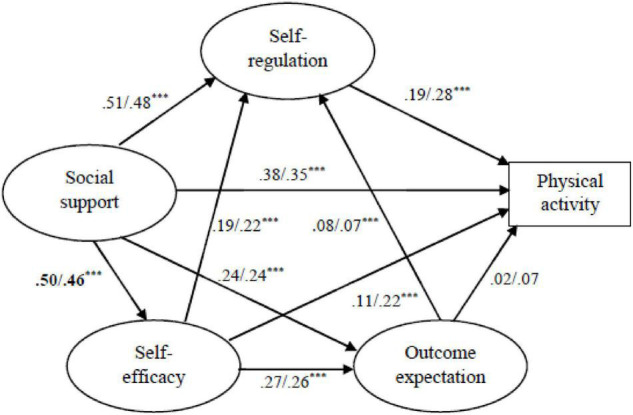
The multigroup comparison of SEM across urban and suburban. Path coefficients are reported male/female subgroup. *** indicates *p* < 0.001, otherwise *p* > 0.05. Bold indicates significant differences of the path coefficients (CRs > 1.96).

The SEM was tested in male and female groups. The hypothesized model was a good representation of the data in both groups, as shown in Table 5. Given that the model offered a good fit for both male and female groups, a multi-structural equation model with latent variables was carried out to study in-depth which parameters could be considered invariant across gender. Table 6 presents the fit indices of the constrained models and the different tests of these models with the unconstrained model. The RMR, GFI, AGFI, TLI, CFI, and RMSEA indicate excellent model fit. The differences between the constrained and unconstrained models indicate that all models appear to be significantly different in all cases at the 1% level. Thus, Model 1, the unrestricted model, was used to assess the path coefficients in the two groups (Figure 3). The architecture of the social-cognitive variables on PA was tested to determine whether there were differences between the urban and suburban groups. After comparing CR for differences between the parameters, the only significant difference was that social support has a more substantial impact on the self-efficacy of male adolescents than on female adolescents. There was no difference in path regression from social cognitive determinants to PA for gender.

**TABLE 5:** The goodness of fit index.

	N	RMSEA	RMR	GFI	AGFI	TLI	CFI	PGFI	PNFI
Male	1191	0.041	0.029	0.976	0.963	0.979	0.984	0.632	0.730
Female	1290	0.049	0.031	0.970	0.953	0.967	0.976	0.619	0.713

**TABLE 6:** Nested Models of Multigroup SEM across male and female.

Model	Chi^2^	df	Chi^2^/df	*p*	RMR	GFI	AGFI	TLI	CFI	RMSEA	Δ Chi^2^	Δ *p*	Δ TLI
**Gender**													
Model 1	531.63	136	3.91	<0.01	0.03	0.97	0.95	0.97	0.98	0.03	−	−	–
Model 2	559.57	149	3.76	<0.01	0.03	0.97	0.96	0.97	0.98	0.03	27.94	0.01	<0.01
Model 3	578.68	155	3.73	<0.01	0.04	0.97	0.96	0.97	0.98	0.03	47.05	< 0.01	<0.01
Model 4	595.60	159	3.74	<0.01	0.05	0.97	0.96	0.97	0.97	0.03	62.97	< 0.01	<0.01
Model 5	670.26	173	3.87	<0.01	0.05	0.96	0.96	0.97	0.97	0.03	138.63	< 0.01	<0.01

## Discussion

This study investigated the predictability of SCT among the Chinese adolescents. The overall model explained 38.9% of the variance in PA. Social support had the greatest effect, self-regulation exerted a moderate effect, and self-efficacy and outcome expectations had a small effect on PA. Negligible differences between the constrained and unstrained models proved the invariance of the SCT model across urbanization and gender. The findings indicate that the SCT model applies to various subgroups and should be considered comprehensively when designing interventions. Specifically, social support had a more substantial impact on the PA of adolescents in suburban areas and urban areas, whereas self-regulation had a more substantial impact on the PA in urban areas than in suburban areas. No difference in path regression from social cognitive variables to PA was found across genders.

Two studies conducted in China used the SCT model to predict the exercise frequency of breast cancer survivors (Hsu H. T. et al., 2011) and obesity prevention behaviors (e.g., TV watching, water consumption, fruits and vegetables consumed, and PA) of children (Murnan et al., 2006). To the best of our knowledge, ours is the first article to investigate the predictive power of SCT to PA among Chinese adolescents. Consistent with studies in other countries (Gao, 2012; Dewar et al., 2013; Bagherniya et al., 2015), we found that the SCT model can predict the PA of Chinese adolescents, which confirmed the first hypothesis. The overall model explained 38.9% of the variance in PA, which can be assessed as substantial (Cohen, 1988; Cohen et al., 2013). The results indicate that the intervention based on SCT could effectively change the PA behavior among the Chinese adolescents. In the case of self-efficacy and PA behavior, although previous studies found self-efficacy as a significant predictor of PA (Gao, 2012; Dewar et al., 2013; Lee et al., 2018), the association between PA and self-efficacy was weak in our study. However, social support was the most critical factor for predicting the PA behavior among Chinese adolescents. The following factors could explain this finding: First, most adolescents in China are engaged in their academic schedule and cannot allocate their time to participate in PA by themselves though they have high self-efficacy. Second, self-efficacy has different categories (e.g., barrier self-efficacy; proxy self-efficacy; and support self-efficacy), which may confine the consistency of the results. In addition, due to the collectivist Chinese culture, their behavior may not be in the control of an individual. They often participate in PA with friends or classmates and have PA scheduled by parents. Further studies that explore the types and sources of social support relevant to adolescents are recommended.

Self-regulation had a moderate effect on PA. Self-regulation is an important set of strategies that could help students gain control over the PA behavior, indicating that individuals need improved goal setting and planning skills to engage in PA. Self-regulation was shown to be a key to success in exercising and adherence to an exercise program, preventing unwanted behavioral tendencies, and focusing on the control of PA behavior (Bandura et al., 1997). Therefore, self-regulation could play a major role in achieving exercise goals, as it serves as an important psychological factor for promoting the positive health behavior, including exercise (Bandura, 2005). This finding has supported the results of studies that found high self-regulation ability predicted the exercise behavior (Anderson et al., 2006; Dishman et al., 2014; Gerber et al., 2015) and was consistent with the finding that people with high self-regulation abilities participated in the PA for more extended periods (Stadler et al., 2009).

Previous studies did not find any relationship between outcome expectations and PA in children (Rovniak et al., 2002; Gao et al., 2009). Our findings found that outcome expectancy had a small effect on the PA behavior, which might be due to the following reasons: first, the adolescents may not sufficiently realize the harmful consequences of physical inactivity, but they recognize the positive social, physical, and psychological expectations of being physically active. Outcome expectancy was reported to be a better predictor of PA among older adults than younger adolescents (Anderson-Bill et al., 2011b; Kosteli et al., 2016). Second, positive PA outcome expectancy of adolescents may not directly translate to the PA behavior (Hankonen et al., 2017). Thus, exploring the additional constructs impacting the relationship between outcome expectancy and PA may be warranted in the future.

Measurement invariance testing for SCT has been less studied. Most researchers would assume that the instruments operate the same way and contain the same constructs across groups. However, the invariance analysis across groups is a logical prerequisite for conducting multigroup comparisons (Vandenberg and Lance, 2000). If not tested, violations of measurement equivalence assumptions threaten substantive interpretations, as it shows the inability to demonstrate reliability and validity (Vandenberg and Lance, 2000). Our findings indicate that the SCT model has the same construct with items associated equally with the factors for the subgroups of urbanization and gender, which indicated the invariance of SCT across urbanization and gender for PA. Future studies could compare the SCT model across different urbanization and gender subgroups.

Social support and self-regulation were found to differ in urban and suburban subgroups, which confirmed our third hypothesis. Different conclusions were obtained in previous studies concerning the predictability of social support to PA. Laird et al. (2016) found that social support did not predict PA in adolescent girls, even though parents and friends may enhance the PA behavior (Laird et al., 2016). However, Martin’s study found that social support is one of the best predictors of PA among underserved middle school students but did not compare the differences between urban school students with underserved school students (Martin et al., 2011). The findings of our study show that the association between social support and PA was higher in suburban than urban areas. The results indicate that Chinese adolescents in suburban areas might not receive sufficient support from their parents and friends for PA, which may be a restricted factor. The association between self-regulation and PA was higher in urban than in suburban areas, which may be explained by the higher levels of social support, quality of PA environments, and quality of PA equipment that adolescents in urban areas receive and have access to. Therefore, the strategic use of skills to remind, cue, or reinforce PA behavior may be more related to PA in urban areas than suburban areas.

The results largely confirm the second hypothesis that there are no differences in the predictive power of SCT on PA behavior in different gender subgroups. This study found no difference between the predictive power of social-cognitive variables to PA across gender subgroups, consistent with previous research (Martin and McCaughtry, 2008; Chen et al., 2019). Results show that the social-cognitive determinants are positively related to PA for boys and girls. One study found that boys were more active and received more support from siblings than girls. The remaining variables did not vary with gender (self-efficacy, parental social support, and friend social support) (Martin and McCaughtry, 2008). Other studies have conflicting results regarding the association between gender and social support. One study found that gender did not moderate the relationship between social support and PA in the Chinese adolescents (Chen and Dai, 2016); however, another reported that boys in primary school were more physically active when a parent praised them for being physically active (Ah Hong et al., 2017), and girls perceived more social support than boys in PA in middle school (Martin and Smith, 2002). Future research should fully explore the relationship between different social support and adolescent PA.

Thus, the SCT model can predict the PA of Chinese adolescents effectively. SCT model could apply to various subgroups to predict the PA behavior and should be considered comprehensively when designing future interventions. However, results from this investigation should be viewed in light of certain limitations. Although the study used Bandura’s classical SCT model, additional relevant PA determinants were not included (e.g., school, peer, and sibling support for PA, barriers, and sociostructurally factors). Second, the self-report questionnaires may have inconsistencies with actual experiences or social desirability bias. Third, given the correlational design of the study, causality cannot be argued. Our findings proved the predictability of SCT on PA among Chinese adolescents, especially the importance of social support and self-regulation. The study investigated the invariances of an SCT model and the difference in the architecture of the social-cognitive variables across urbanization and gender subgroups. The findings indicate that future possibilities for promoting PA interventions among Chinese adolescents should include those variables. However, a questionnaire was used to measure PA due to the large sample size in this study. Future studies should consider measuring PA behavior using accelerometers that can objectively measure PA. Furthermore, future studies should look beyond SCT models and use these in conjunction with broader social-ecological models that incorporate determinants at multiple levels (policy and environment), which could further understand determinants of PA participation among adolescents (Sallis and Owen, 2015).

## Data Availability Statement

The original contributions presented in the study are included in the article/supplementary material, further inquiries can be directed to the corresponding author.

## Ethics Statement

The studies involving human participants were reviewed and approved by the Ethical Committee of the Medical Association of Tsinghua University approved the study (IRB#20190093). Written informed consent to participate in this study was provided by the participants’ legal guardian/next of kin.

## Author Contributions

XM contributed to the idea and supervision. JL wrote the original draft preparation and edited the manuscript. MZ analyzed and interpreted the data. YZ analyzed part of the data. DW and BS revised the manuscript. All authors have read and agreed to the published version of the manuscript.

## Conflict of Interest

The authors declare that the research was conducted in the absence of any commercial or financial relationships that could be construed as a potential conflict of interest.

## Publisher’s Note

All claims expressed in this article are solely those of the authors and do not necessarily represent those of their affiliated organizations, or those of the publisher, the editors and the reviewers. Any product that may be evaluated in this article, or claim that may be made by its manufacturer, is not guaranteed or endorsed by the publisher.

## Figures and Tables

**APPENDIX FIGURE 1:**
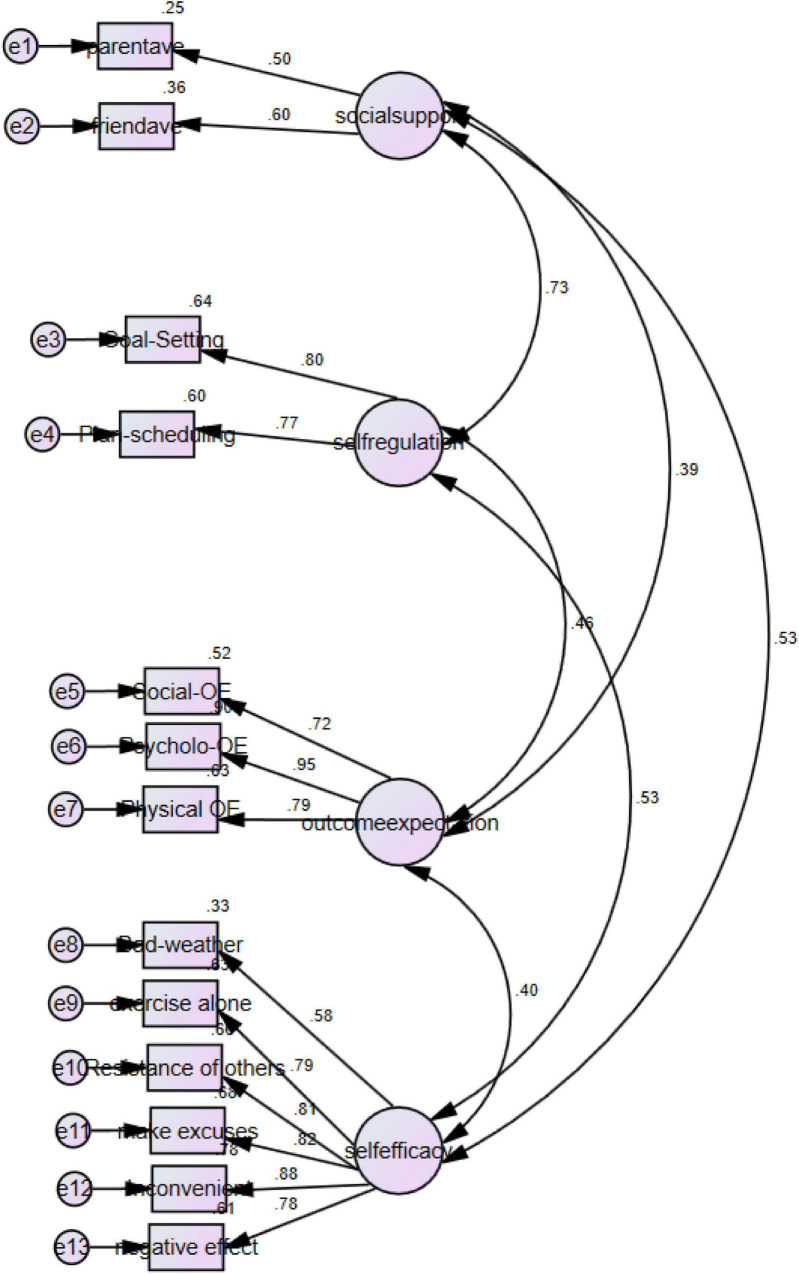
The confirmatory factor analysis (CFA) of the model and the validity of scales in different dimensions.
